# Improved Metal Cation Optosensing Membranes through the Incorporation of Sulphated Polysaccharides

**DOI:** 10.3390/molecules27155026

**Published:** 2022-08-07

**Authors:** P. R. M. Santos, A. Johny, C. Q. Silva, M. A. Azenha, J. A. Vázquez, J. Valcarcel, C. M. Pereira, A. F. Silva

**Affiliations:** 1 Research Center in Chemistry UP (CIQUP), Institute of Molecular Sciences (IMS); Departamento de Química e Bioquímica, Faculdade de Ciências da Universidade do Porto, Rua do Campo Alegre, 4169-007 Porto, Portugal; 2 KAUST Catalysis Center, Catalysis Nanomaterials and Spectroscopy (CNS), King Abdullah University of Science and Technology, Thuwal 23955, Saudi Arabia; 3 Grupo de Reciclado y Valorización de Materiales Residuales (REVAL), Instituto de Investigaciones Marinas (IIM-CSIC), C/Eduardo Cabello, 6, 36208 Vigo, Spain

**Keywords:** membranes, sulphated polysaccharides, biopolymers, optosensors, surfactant, CTAB, aluminum cation

## Abstract

Optosensing chitosan-based membranes have been applied for the detection of heavy metals, especially in drinking water. The novelty of this study is based on the use of sulphated polysaccharides, in such optosensing membranes, aiming at an improved analytical performance. The sulphated polysaccharides, such as ulvan, fucoidan and chondroitin sulfate, were extracted from by-products and wastes of marine-related activities. The membranes were developed for the analysis of aluminum. The variation in the visible absorbance of the sensor membranes after the contact between the chromophore and the aluminum cation was studied. The membranes containing sulphated polysaccharides showed improved signals when compared to the chitosan-only membrane. As for the detection limits for the membranes containing ulvan, fucoidan and chondroitin sulfate, 0.17 mg L^−1^, 0.21 mg L^−1^ and 0.36 mg L^−1^ were obtained, respectively. The values were much lower than that obtained for the chitosan-only membrane, 0.52 mg L^−1^, which shows the improvement obtained from the sulphated polysaccharides. The results were obtained with the presence of CTAB in analysis solution, which forms a ternary complex with the aluminum cation and the chromophore. This resulted in an hyperchromic and batochromic shift in the absorption band. When in the presence of this surfactant, the membranes showed lower detection limits and higher selectivity.

## 1. Introduction

With population growth there is an increase in food waste production, since a large part of the by-products generated by contemporary food remains underused, despite that these often contain high-value substances. A crucial problem faced by industries and society during food processing is, thus, food waste disposal.

From the marine resources that are wasted, it is possible to extract several biopolymers, among which are: ulvan (Ul)—from green algae; chondroitin sulfate (CS)—from the cartilage of sharks and other fish; fucoidan (Fu)—from brown algae; and chitosan (CH)—extracted mainly from the shells of crustaceans. In the case of chitosan, it has been estimated that about 1011 tons of chitin, a precursor of chitosan, are produced annually by living organisms in the ocean [1], opening huge possibilities for applying this biopolymer. Chitosan has been commonly used for the preparation of several adsorbents for removal of pollutants from aqueous environments [2].

The use of biopolymers, more specifically biodegradable and biocompatible polysaccharides, has become very attractive [2,3,4]. Due to their availability and relatively low cost, chitosan and sulphated polysaccharides, such as chondroitin sulfate, fucoidan and ulvan, among others, are considered materials compatible with the circular economy [5]. They exhibit some interesting properties that are worth exploring. Due to the presence of negatively charged groups, these polysaccharides are known to interact with cations, especially with metal cations [6,7,8,9,10]. This can represent an asset to the development of new materials for detection, extraction or separation of heavy metals, which are known to be prejudicial to the environment and health of living beings.

One of the applications of these biopolymers, most specially chitosan [11,12,13,14], has been the preparation of optosensing membranes for metal cations. The polymers that make up the membrane not only act as the solid supports, on which the recognition elements are immobilized, but can also provide permeation selectivity for certain species while rejecting others.

There are different optosensing membranes described in the literature for detection of metals that contain polysaccharides in their structure, making use of different optical properties for detection, such as the refractive index, resonance angle and colorimetric properties [15,16,17,18,19,20]. Metal cations can be determined using complexing reagents, also known as chromophores. The color of the formed complex differs from that of the complexant species and/or the metal cation, since the binding reaction with the ions is accompanied with a change in light-absorption properties. There is the appearance of an absorption band with the simultaneous disappearance of another, while in other cases, there would only be a change in the intensity of a single band [21].

It is possible to find some reports on optosensing membranes for metals containing chitosan, based on colorimetric changes. The synthesis of a thin film of chitosan that incorporates quercetin for the colorimetric detection of Al(III) is described. The complexation of quercetin, immobilized onto the thin film with Al(III), leads to a change in absorbance, which translates to a change in color, going from colorless to orange [22]. In another article, the production of a membrane based on chitosan and polysiloxane is described, to which dithizone was immobilized in order to detect the presence of Pb(II) in aqueous solutions. This optical sensing membrane showed a detection limit of 110 µg L^−1^ [13]. Other membranes containing chitosan were prepared, such as a chitosan/cellulose membrane, functionalized with rhodamine in order to change color in the presence of a metal cation [11]. In this case, the colorimetric changes in the presence of Hg(II), Cu(II) and Zn(II) were studied. The membrane produced different colors in the presence of the different metal cations; however, the changes were not easily visible through the naked eye. In a solution with all the different metal cations, the mutual interferences did not allow for the determination of the presence or quantification of any of them. A chitosan membrane incorporating disodium-1-nitroso-2-naphthol-3,6-disulfonate or nitroso-R was also described in the literature. The membrane changed its color in the presence of Co(II), going from yellow to bright red. This allows one to measure the absorbance of different standards and determine a detection limit of 12 µg L^−1^ [12].

These optosensing membranes showed detection limits ranging from 0.6 µg L^−1^ to 1112 µg L^−1^; however, most of them are not proven to be selective to the metal cation chosen. From the different optosensing membranes presented in the literature, none made use of the sulphated polysaccharides. All these membranes made use of chitosan, probably due to its viscosity that leads to an easy and fast formation of a membrane, contrarily to sulphated polysaccharides. Although, the use of a combination of chitosan and chondroitin sulphate for the preparation of a membrane for biomedical applications was reported [23].

Therefore, the literature still lacks reports that explore the use of these compounds to produce optosensing membranes selective and sensitive to a certain metal cation. The choice of polymer material has a determining effect on sensor performance. The response time, for example, depends mainly on the diffusion coefficients of the ions in the polymeric network [24]. In this article, we sought to obtain optosensing membranes comprising sulphated polysaccharides and their application to the detection and quantification of aluminum cation through the immobilization of eriochrome cyanine R (ECR) onto the membrane.

## 2. Results and Discussion

### 2.1. Membrane Synthesis

To apply the sulphated biopolymers to a membrane, it was necessary to test different molar proportions of sulphated polysaccharide: chitosan monomer, in order to maximize the content of chondroitin sulfate, fucoidan and ulvan present in the final membrane. For that purpose, proportions from 1:210 to 1:10 were tested. The maximized proportion determined was around 1:40. Higher proportions led to the formation of precipitates before casting at the Petri dish.

Since the membranes presented some problems in terms of mechanical properties, more specifically their instability in solution, owing to high swelling degrees (850–950% *Sw*), it was necessary to resort to a crosslinking agent, namely glutaraldehyde, to increase the cohesion in the material. A study on the addition of this compound was carried out, testing different molar proportions of glutaraldehyde to chitosan monomer, between 0:1 and 1:150. 

The determination of the swelling degree is important, as it allows one to evaluate the ability of the membrane to interact with the aqueous solution. If the swelling is practically null, little or no solution, which will contain the analyte of interest, will be in contact with the internal functional groups. In addition to this factor, it provides other information about the stability of the membrane immersed in water. High degrees of swelling (in the order of 900% *Sw*) caused the membrane to rupture easily when agitation was applied.

In the case of CH-PSil (chitosan-polysiloxane) membrane, proportions of 1.5:1, 1:3, 1:15, 1:50 and 1:150 mols of glutaraldehyde to mols of chitosan were tested. From all the proportions studied, 1:50 gave the best combined results, reducing the swelling to 500–550% *Sw*, and giving acceptable malleability. A higher proportion of glutaraldehyde (>1:50) led to very rigid and brittle membranes with a very low degree of swelling (<20%) and a red tint. A smaller proportion of glutaraldehyde translated to higher degrees of swelling.

In the case of membranes containing sulphated polysaccharides, similar proportions were tested and the selected ratio was 1:30.

Although glutaraldehyde improved the stability of the membrane, it made it a little more rigid. Since the membranes would break after bending them a few times, glycerol was added, which works as a plasticizer in the presence of biopolymers to improve the malleability. Different glycerol:chitosan monomer molar proportions were tested, 1:70, 1:7, 1:2 and 1:1. Initially, very small amounts of glycerol were tested, something that resulted in the unaltered malleability of the membrane. As the amount of glycerol incorporated in the membrane was gradually increased, an improvement in the malleability began to be noticed, until at a ratio of 1 mol of glycerol to 1 mol of chitosan monomers, it was possible to reach the desired malleability. In the case of CH-CS, CH-Fu and CH-Ul membranes, a similar test was performed and the chosen ratio was 2:1.

### 2.2. Evaluation of Membrane Composition 

**Figure 1:**
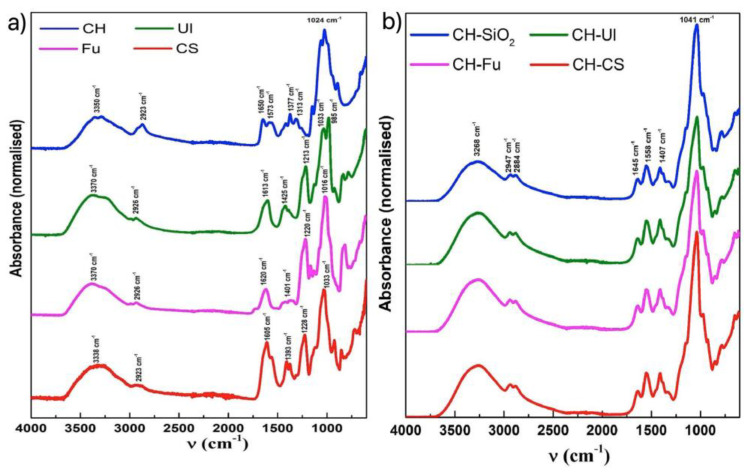
Graphical representation of ATR-FTIR spectra of: (**a**) different polysaccharides; (**b**) different types of membranes.

The composition of the prepared membranes was analyzed using Fourier Transform Infrared Spectroscopy coupled with attenuated total reflectance (ATR) module. For confirming the presence of the expected components, ATR-FTIR spectra of CH, Ul, Fu and CS samples were studied for reference. Figure 1a illustrates the ATR-FTIR spectra for the individual components..

From the literature, it is possible to identify characteristic bands of the different polysaccharides. Broad bands in a range of 3200–3500 cm^−1^, due to stretching vibrations of O-H bonds and C-H stretching bands around 2926 cm^−1^, are common to all four spectra [25,26]. Chitosan shows two characteristic bands at 1650 cm^−1^ and 1573 cm^−1^, indicating C=O stretching band of the acetyl group (amide I) and -NH bending and stretching (amide II) bands, respectively [26]. The stretching vibrations of S-O bonds at 1220 cm^−1^ and the carboxylic peaks at about 1620 cm^−1^ and 1400 cm^−1^ are typical of sulphated polysaccharides, such as CS, Fu and Ul [27]. In addition, the bands at 1016 cm^−1^ to 1033 cm^−1^ indicate C-O-C stretching vibrations and bands around 980 cm^−1^ represent asymmetrical stretching vibration of C-O-S bonds, while the signals in a range of 822 cm^−1^ to 849 cm^−1^ were assigned to the symmetrical stretching vibrations of C-O-S bonds [28].

The FTIR spectra for the different types of chitosan-based membranes (Figure 1b) present the major characteristic bands found in the individual compounds. It is possible to verify the presence of silica from the peaks at around 1041 cm^−1^. In addition, the appearance of characteristic peaks at 1558 cm^−1^ and 1645 cm^−1^ marks the presence of chitosan in all the membranes. However, unequivocal identification of Ul, Fu and CS in the membranes was not possible due to (i) the fact that the sulphated polysaccharides represent only 2.3% of the respective membrane mass and (ii) the fact that their distinctive bands are on the same wavenumber ranges, thus, overlapped with the peaks from the major components.

As a way of complementing the composition evaluation, more specifically the presence of sulphated biopolymers, a second technique was used, namely thermogravimetric analysis (TGA). It was possible to obtain the thermogravimetric curves (Figures S1–S3) for the different membranes without the addition of glycerol, since this is a major component with a boiling point at 290 °C and could mask the presence of other biopolymers that were added at 2.3% in mass only. As expected, there was no degradation of silica in the analyzed temperature range, since it only degrades from 1000 °C [29]. In the case of chitosan, when it was incorporated into the membrane, it became less thermally stable, starting to degrade at a lower temperature, around 230 °C, compared to the biopolymer in its pure state, which starts at 300 °C. In the literature, it is reported that in the TGA of pure chitosan, from 30 to 150 °C, there is dehydration, while around 330 °C, there is a maximum mass loss [30]. The value obtained differs a little from the literature, possibly due to different methods of extraction of chitosan and different degrees of purity. 

When analyzing the membranes containing the sulphated polysaccharides, the temperature of degradation for the sulphated polysaccharides was similar to the chitosan in the membranes, which made the separate quantification of each polysaccharide impossible.

Although it was not possible to determine the percentage of each polymer, the theoretical values expected to be in the membranes without glycerol and glutaraldehyde are 54% of chitosan in CH-PSil membrane and 56% of total polysaccharides in the membranes with sulphated polysaccharides. From the TGA analysis, it was possible to conclude that chitosan represented 58–60% in CH-PSil membrane and total polysaccharides represented 62–64% in the other membranes. The increase in percentage of mass of total polysaccharides degraded, compared with the CH-PSil membranes, allows one to conclude that the sulphated polysaccharides were present in the membrane, since, theoretically, the addition of the sulphated polysaccharides would translate into an increase of 2%.

The thickness of the membrane was also determined, as it is essential for the ionic diffusion through the membrane, since the thicker it is, the longer it will take for the ions to diffuse through the membrane. The thickness was controlled through the volume of deposition, testing different deposition volumes from 6 to 15 mL. The minimum thickness achieved was 50 ± 10 µm with 8 mL of deposition for the membrane CH-PSil, due to being the minimum volume to cover the Petri dish. To achieve the same thickness, for the sake of result comparability, in the case of the membranes with sulphated polysaccharides, 10 mL of the casting solution were needed instead.

### 2.3. Study of the Incorporation of ECR

**Figure 2:**
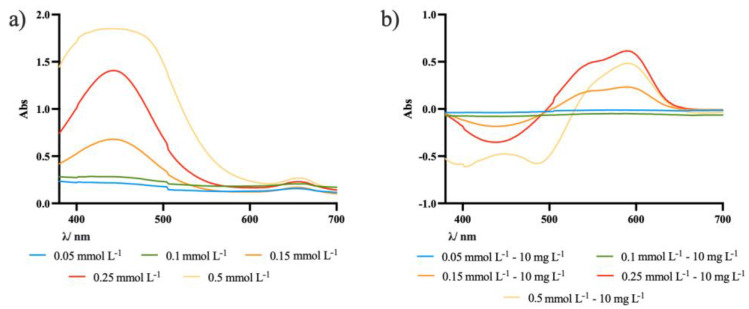
Graphical representation of UV-Vis spectra of: (**a**) CH-Ul membrane with different concentrations of ECR; (**b**) CH-Ul membranes after immersion on 10 mg L^−1^ Al(III) solution.

The successful incorporation of a chromophore into the membrane is a crucial step in obtaining a sensing membrane. The incorporation of ECR into the membrane was studied by immersing the membrane in solutions with different ECR concentrations for 24 h (Figure 2a).

Afterwards, the membranes with different concentrations of ECR were immersed in a solution of 10 mg L^−1^ of Al(III) (Figure 2b).

The ECR-immobilized membranes in the absence of Al(III) had a characteristic absorption peak at around 450 nm. Upon complexation on exposure to Al(III) ions, the membranes changed color from yellow to violet, resulting from the appearance of new bands at higher wavelengths, more specifically at around 545 nm and 590 nm.

From the analysis of the graphical representation of the UV-Vis spectra, it is possible to conclude that the best signal was given for the membrane immersed in the 0.25 mmol L^−1^ solution. Above that concentration, the absorbance of the spectrum that will be taken as background spectrum is too high, becoming an unstable reference (see also Figure S1).

### 2.4. Study of Immersion Time on the Cation Solution

For the application of the developed membrane incorporated with ECR as an optical sensing platform for the detection of Al(III), it was necessary to optimize the immersion time in the aluminum-cation-containing solution, prior to optical analysis. In order to verify the optimal contact time of each membrane with the solution containing Al(III) ions, all the four types of membranes prepared were subjected to kinetics studies, separately, by immersion in 10 mg L^−1^ Al(III) in buffer solution of pH 5.5 and analyzed using UV-Vis spectroscopy at regular time intervals (Figure S4). 

**Figure 3:**
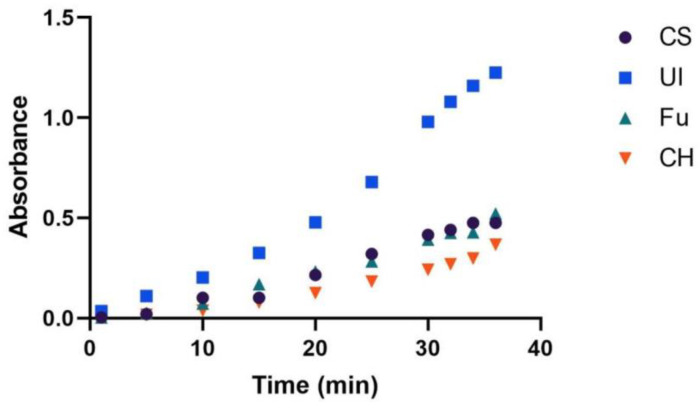
Variation in the absorbance as a function of time in different types of membranes.

From the plot given in Figure 3, it can be inferred that the increase in the immersion time allows a greater number of aluminum ions to bind to ECR, forming an Al(III)-ECR complex. However, it is important to choose an optimal time of immersion, which can give good optical signals for sensing applications. Despite not all signals stabilized after 30 min, this time was set as a compromise between sensitivity and analysis time, but it had to be tightly controlled.

### 2.5. Evaluation of the Membrane Performance

**Figure 4:**
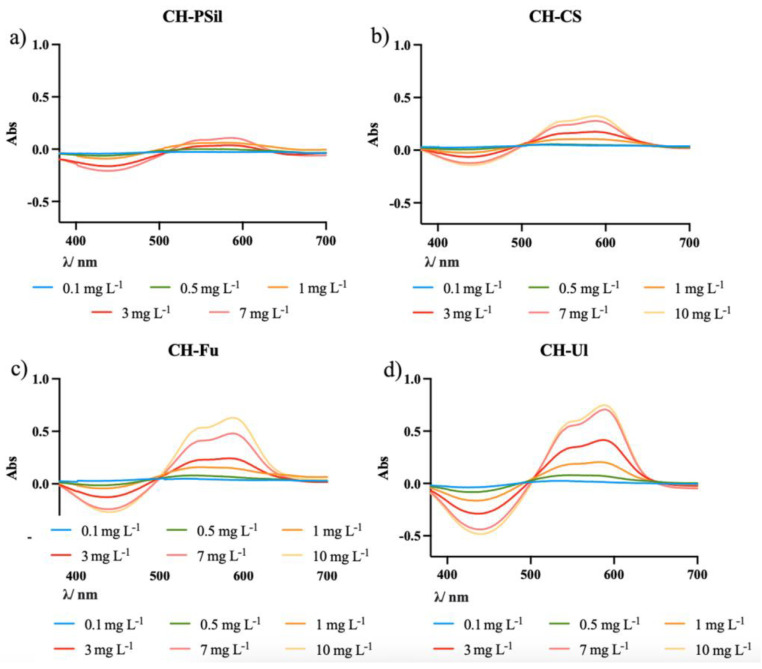
Graphical representation of absorption spectra for the different types of sensing membranes in solutions with different Al(III) concentrations: (**a**) CH-PSil; (**b**) CH-CS; (**c**) CH-Fu; (**d**) CH-Ul.

The performance of the sensing membranes was evaluated using the spectral data obtained by UV-Vis spectroscopy after 30 min of immersion in different concentrations of Al(III) in the range of 0.1 mg L^−1^ to 5 mg L^−1^. Figure 4 shows the absorption spectra for the different types of sensing membranes.

The study of the membranes containing chondroitin sulfate was conducted with the several different CSs available, three from marine origin, extracted from different species, and one commercial bovine CS. However, the following results pertain to the commercial CS only, since any significant influence of the CS source in the membrane properties was not observed. 

**Figure 5:**
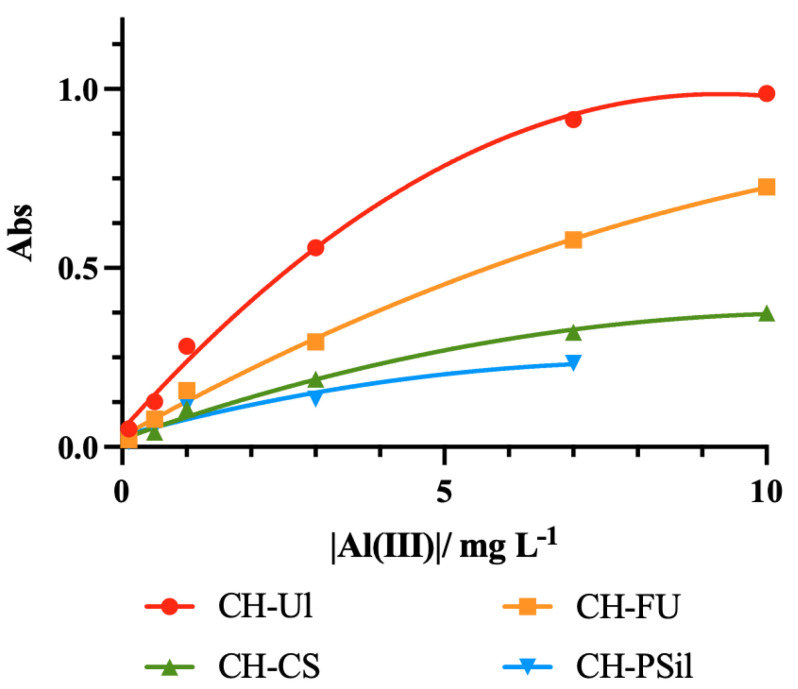
Calibration curve of the signal for the different membranes in 0.1 to 10 mg L^−1^ of Al(III).

There are two absorption maxima at around 545 nm and 590 nm. However, since the sensitivity at 590 nm is higher, this wavelength was selected for measuring the response towards Al(III). Similarly, a corresponding decrease in the absorption maxima of ECR at 450 nm can also be observed as the ECR-Al(III) complex formation band represented at 590 nm increases. A calibration curve for the results obtained with the maximum absorbance at 590 nm was produced (Figure 5). 

It is possible to see that none of the membranes displayed a linear response to the presence of the aluminum cation in the range chosen. However, it is also possible to visualize a difference in the response from the different membranes. The CH-PSil membrane was the one with the poorest signal. CS, Fu and Ul possess carboxylate and sulfonate groups, while CH does not. These functional groups interact better with the aluminum cation [6,7,8], allowing a greater quantity of Al(III) to easily permeate the membrane, leading to the formation of a higher amount of ECR-Al(III) complexes, which translates into higher absorbances. In the absence of such functional groups, the Al(III) will not permeate the membrane so easily, forming mostly complexes with the ECR present on the surface. The CH-Ul membrane exhibited the highest signal response. From the information provided by the supplier, the extraction process of Ul required several steps of purification for the removal of aluminum from its naturally available form. This shows that ulvans, by nature, have strong interactions with Al(III) and, as mentioned above, the cation will easily permeate the membrane to meet the chromophore.

### 2.6. Study of Surfactant Incorporation

ECR is a good spectrophotometric reagent for the detection of Al(III) ions [31]. However, the spectrophotometric methods for the determination of aluminum based on binary complexes with triphenylmethane reagents, such as ECR, exhibit moderate sensitivity. The introduction of a third component (a long chain quaternary base (cationic surfactant)) to the binary system, leads to the formation of a ternary complex with advantageous properties. Spectrophotometric methods based on such ternary systems are expected to be more sensitive than the ones based on binary systems for the detection of metal ions [32]. Further, the expected red shift in the peak wavelength can give beneficial effects in terms of avoiding interferences. Hence, the effects of incorporation of the cationic surfactant CTAB on the sensitivity of ECR-Al(III) complexation on the membrane in an acetate buffer environment (pH 5.5) were investigated in this study.

CTAB’s incorporation into the membrane during the synthesis was initially attempted in two different molar ratios (1:1 and 1:2 ECR:CTAB). However, no positive enhancement was observed in the performance of sensing membranes in comparison to the results obtained without the use of a surfactant. 

**Figure 6:**
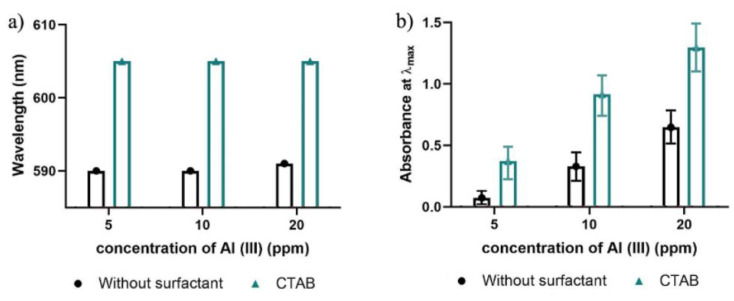
Effect of incorporation of 2 mL CTAB at 1 mmol L^−1^ into the Al(III) test solution, demonstrated by the variation in (**a**) peak wavelength and (**b**) absorbance intensity as a function of concentration of Al(III) solution.

The second approach was the addition of the surfactant after the synthesis of the membrane. This was achieved by adding the surfactant to the sample solution to be analyzed, according to the literature [33]. The results of the peak position and measured absorbance are shown in Figure 6.

It is evident from the graph that the incorporation of CTAB could significantly enhance the sensitivity of the sensing membrane. There was a red shift from around 590 nm to 605 nm. The absorption maxima at 590 nm corresponds to the binary ECR-Al(III) complex, whereas the absorption maxima at 605 nm stands for the ternary ECR-Al(III)-CTAB complex formation. The color change produced in this case was yellow to blue. This happens due to the high capacity of the surfactant to solubilize an insoluble complex and/or ligands by micellar solubilization or formation of ternary complexes containing surfactant monomers, which leads to an improvement in sensitivity (molar absorptivity) and red shift [34].

**Figure 7:**
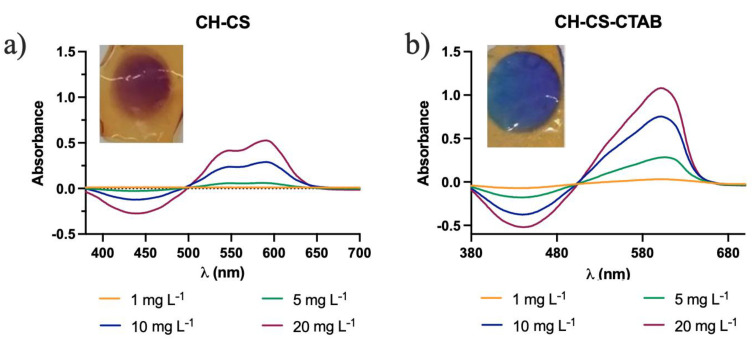
Graphical representation of the UV-Vis spectra and respective image of the CH-CS membrane after immersion in different concentrations of Al(III): (**a**) without CTAB; (**b**) with CTAB.

Figure 7 exemplifies the change in color observed in a CH-CS membrane with and without the use of CTAB and in its corresponding spectra. 

Since the incorporation of CTAB was found to be advantageous, the effect of CTAB on the optical absorption behavior of all the four membranes was studied in a range of 0.1 to 5 mg L^−1^ with the addition of 2 mL of 1 mmol L^−1^ CTAB. The spectra had similar profiles to the one represented in Figure 7b (Figure S5). 

**Figure 8:**
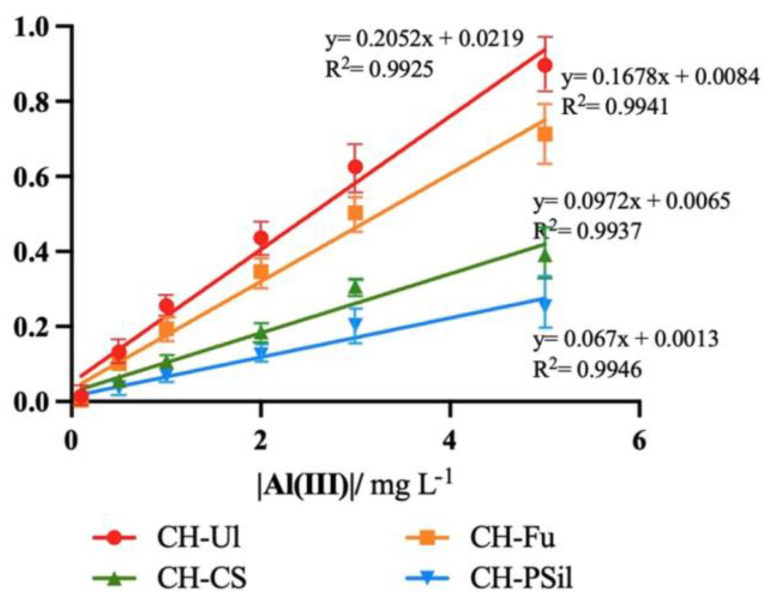
Calibration curves with the different membranes for 0.1 to 3 mg L^−1^ of Al(III) with CTAB.

While the analysis without CTAB showed two overlapped bands, one around 545 nm and another one, more intense, at 595 nm, with CTAB, only one peak is shown, at around 605 nm. Analyzing the maximum at the peak of 605 nm for each concentration made it possible to build a calibration curve (Figure 8).

With the addition of CTAB, it was possible to obtain a linear calibration in a range 0.1 to 5 mg L^−1^. Then, the detection limits were determined. For the CH-PSil membrane, a detection limit of 0.52 mg L^−1^ was obtained. In the case of the CH-CS membrane the detection limit was 0.36 mg L^−1^. The CH-Fu and CH-Ul membranes showed the best results, with a detection limit of 0.21 and 0.17 mg L^−1^, respectively. The CH-Ul membrane represented an improvement in the detection limit of about five-times compared to the chitosan-only membrane, which presented the highest detection limit value. The introduction of sulphated polysaccharides, in all cases, led to an improvement in Al(III) detection.

### 2.7. Study of the Effect of Interfering Species on the Selectivity of Sensing Membranes

In practice, no sensing membranes have 100% selectivity to a particular metal ion. The performance is likely to be affected by interfering species. Based on the literature [35], various relevant cationic and anionic species likely to interfere with Al(III), such as Cu(II), Fe(II), Fe(III), Cd(II), Pb(II), Cr(VI) and F^−^, were selected, as they were known to show interference with the determination of Al(III) with ECR in the solution phase. In the membrane phase, the influence of these interferents was not known previously. Hence, it was critical to evaluate that influence.

**Figure 9:**
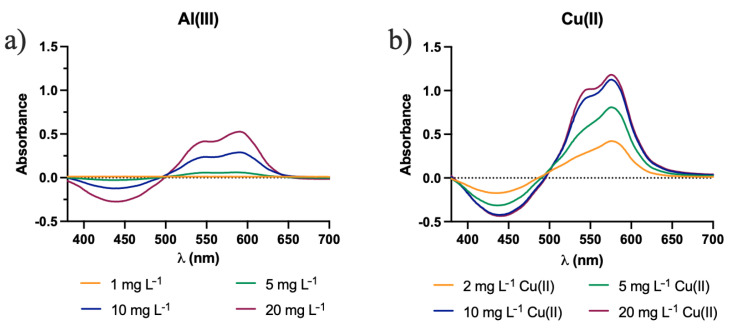
Graphical representation of the UV-Vis spectra of the membrane CH-CS after immersion in different concentrations of: (**a**) Al(III); (**b**) Cu(II).

The study was carried out in CH-CS membranes, both in the presence and absence of CTAB. Initially, the effect of these potential interfering species was individually examined. The spectra obtained when different interfering ions were individually analyzed on the CH-CS membranes without the incorporation of CTAB. Cu(II) exerted a major interference with Al(III) ions, showing a well-defined absorption peak at 574 nm, even at lower concentrations (Figure 9). The other interfering species did not show relevant absorbance (Figure S6) anywhere near the band of the aluminum and so could not interfere significantly in the determination of the Al(III), in a range of 1 to 20 mg L^−1^.

From the spectral data obtained, it is possible to determine the selectivity coefficient (K) for each interfering ion. From the single analyte measurements, admitting an independent behavior for Al(III) and interferent, the selectivity coefficient can be estimated.

**Figure 10:**
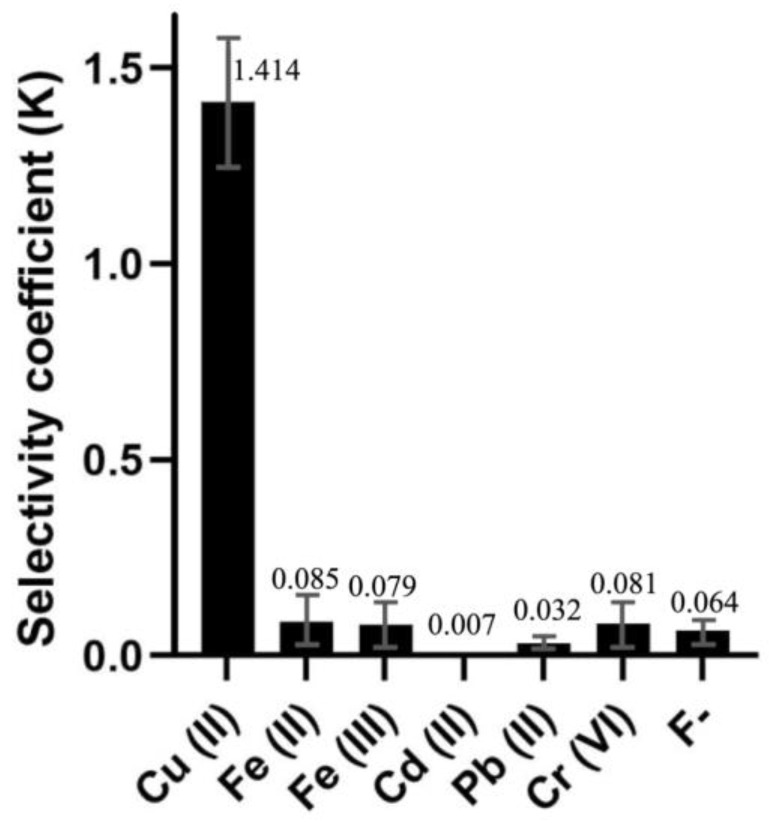
Selectivity data for the different interfering species.

For the analysis of CH-CS membranes without CTAB, all the absorbance values were measured at the peak wavelength 590 nm. The K values obtained for CH-CS membranes in the absence of CTAB are given in Figure 10. The lower the K value, the lesser the interference and more selective the membrane towards Al(III).

From Figure 10, Cu(II) can, as expected, be identified as a severe interference to Al(III) using the membrane under study with almost 1.5-times higher selectivity over Al(III). Cu(II), being an intermediate ligand, has good affinity with ECR and is known to form Cu(ECR)_2_ complexes [36]. However, other factors, such as permeability, ionic radius, etc., might also be contributing factors to the high interference of copper in this proposed Al (III)-sensing membrane. It should also be noted that the K values of other non-interfering species might be overestimated due to the errors in baseline corrections.

**Figure 11:**
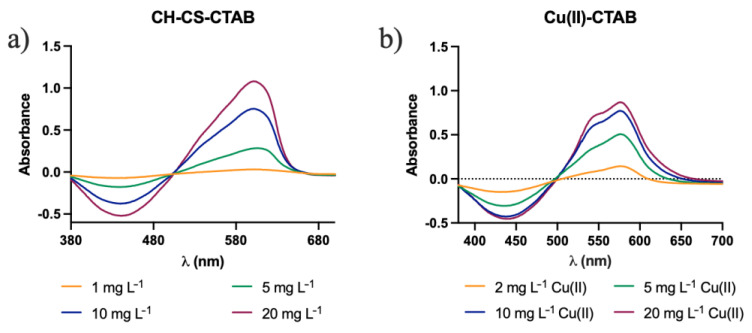
Graphical representation of the absorption spectra of CH-CS membranes after immersion in samples containing CTAB and: (**a**) Al(III); (**b**) Cu(II).

However, the use of CTAB was expected to contribute to the minimization of the effect of interfering species. The spectra obtained when Al(III) and Cu(II) were individually analyzed on the CH-CS membranes in the presence of CTAB are shown in Figure 11.

**Figure 12:**
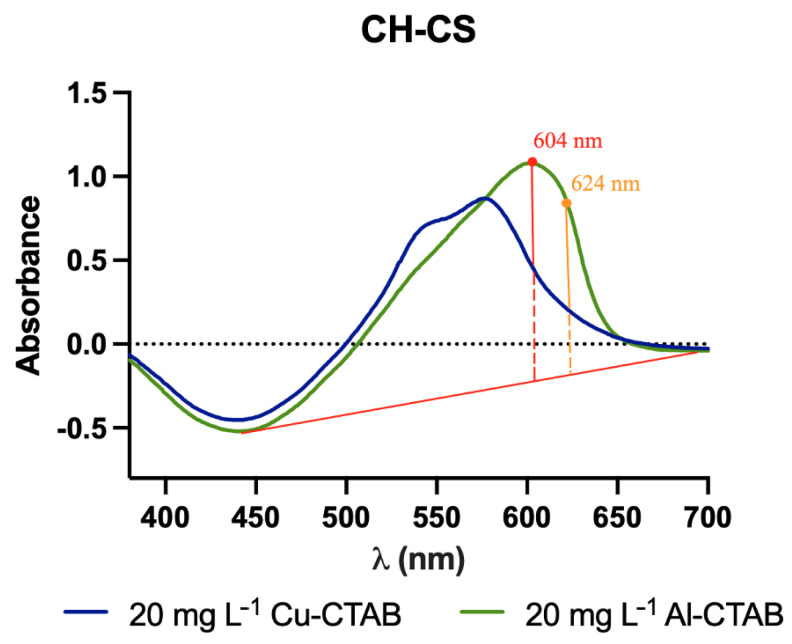
Absorption spectra of CS membranes on immersion in CTAB-supplemented solutions of Al(III) and Cu(II) at 20 mg L^−1^ concentration—comparison between the signals read at 605 nm and 624 nm.

No change in peak wavelength was found (at 575 nm) in the case of Cu(II) complex with CTAB, whereas the Al(III) complex peak wavelength changed from 590 nm to 604 nm in the presence of CTAB (perceivable better at Figure 12). It was observed that the sensitivity of Cu(II) decreased, while that of Al(III) increased upon addition of CTAB. The combined effect of this change in sensitivity along with the red shift for Al(III) upon CTAB addition was expected to minimize the effect of interference from Cu(II) to the proposed Al(III)-sensing membrane. 

The selectivity coefficient K for Cu(II) in CH-CS membranes in the presence of CTAB was estimated to be 0.40, when all absorbance values were measured at the aluminum peak value 605 nm. The new K value for Cu(II) is much lower than the K value obtained for CH-CS membranes without CTAB, indicating lesser interference of Cu(II) in the Al(III)-sensing membrane. It is also notable that the K value for Cu(II) can even be as small as 0.24 at the wavelength 624 nm, though at the expense of the sensitivity.

## 3. Materials and Methods

### 3.1. Materials and Reagents

Ulvan (Ul), extracted from *Ulva lactuca* (molecular weight around 300 KDa) was kindly provided by Stemmaters, Portugal. Chondroitin sulfate (CS) sodium salt, extracted from bovine cartilage was provided by Carbosynth, UK. CS extracted from cat shark (*Scyliorhinus canicula*), CS extracted from blackmouth catshark (*Galeus melastomus*) and CS extracted from rabbit fish (*Chimaera monstrosa*) at the Instituto de Investigaciones Marinas (IIM-CSIC) [37,38,39] were also tested. Fucoidan (Fu) extracted from *Fucus vesiculosus* (92.7%) was provided by Marinova, Australia. Chitosan (CH) (low-molecular weight), glutaraldehyde (70% in water), acetic acid (≥99.7%) and sodium acetate were purchased from Sigma Aldrich, Germany. Metal cation salts such as lead nitrate, cadmium nitrate, copper sulphate, sodium fluoride and ferrous sulphate were supplied by Merck, Germany. Hydrochloric acid (≥37%), anhydrous glycerol (≥99.5%) and aluminum sulphate hexadecahydrate were purchased from Fluka, Switzerland, while eriochrome cyanine R was purchased from Riedel-de Haen, Germany. Nitric acid (65%) and cetyl trimethyl ammonium bromide were purchased from PanReac, Spain. Tetraethylorthosilicate (≥98%) was purchased from Acros Organics, Germany, and absolute ethanol was from Fischer Scientific, UK. Water used was of Milli-Q purity, Millipore, Massachusetts, EUA.

### 3.2. Methods

#### 3.2.1. Synthesis of the Membranes

Herein, the preparation of the four types of chitosan-based sol gel membranes is presented. The procedure followed for the synthesis of membranes was primarily adapted from the one described in the literature [13] with necessary modifications in accordance with the type of membranes prepared. The initial step in the preparation of the tetreaethylorthosilicate (TEOS) solution is the same for all the membranes and is a major step for the formation and incorporation of polysiloxane (PSil) chains by the sol–gel process. For this a solution containing 5.5 mL of TEOS, 5.5 mL of ethanol, 22.2 mL of MilliQ water and 1.25 mL of 0.1 M HCl was prepared and left under moderate magnetic stirring for a period of 24 h.

- Synthesis of Chitosan–Polysiloxane Membranes (CH-PSil)

A 3% (*w*/*v*) CH solution was prepared by dissolving the biopolymer in 2% acetic acid solution. Then 10 mL of that solution was mixed with 5 mL of the already prepared TEOS solution and stirred for 2 h. The next step consisted of the addition of 2 mL of 8% (*w*/*v*) glycerol, which acted as a plasticizer and the solution was kept under stirring for another 2 h. This was followed by the addition of 150 μL of 2.5% (*w*/*v*) glutaraldehyde, the cross-linking agent. After stirring for 1 h, 8 mL of the final solution was deposited into a Petri dish of about 80 mm in diameter and allowed to dry at room temperature.

- Synthesis of Membranes Containing Sulphated Polysaccharide

Three different types of membranes were synthesized by adapting the previous synthesis. This synthesis consisted of mixing 6 mL of 3% (*w*/*v*) CH in 2% acetic acid solution with 6 mL of 0.2% (*w*/*v*) polysaccharide (CS or Fu or Ul) in aqueous solution and left stirring for 2 h. Then 3 mL of the already prepared TEOS solution was added and allowed to stir for 2 h. The next steps were the addition of the plasticizer and the crosslinker according to the previous description. The final step was the deposition of 10 mL of the final solution into a Petri dish of about 80 mm in diameter and allowing to dry at room temperature. 

#### 3.2.2. Membrane Characterization

To carry out the characterization of the membranes, different determinations were used, such as the degree of swelling, malleability and thickness, as well as instrumental techniques such as the FTIR-ATR and TGA.

- Degree of Swelling

To determine the degree of swelling, 100 mg of each membrane was placed in 2 mL microcentrifuge tubes, with 1.5 mL of ultrapure water for 24 h under agitation. After the swelling time, it was centrifuged at 12,000 rpm for 5 min in order to facilitate the removal of water that had not been incorporated into the membrane. The calculation of the degree of swelling was performed using Equation (1):

(1) $$Sw\;\left(\%\right)=\frac{W_{f}-W_{0}}{W_{0}}\times 100$$

where *W*_0_ represents the initial material weight and *W_f_* the final material weight.

The procedure was performed in duplicate for all different membranes.

- Malleability

**Figure 13:**
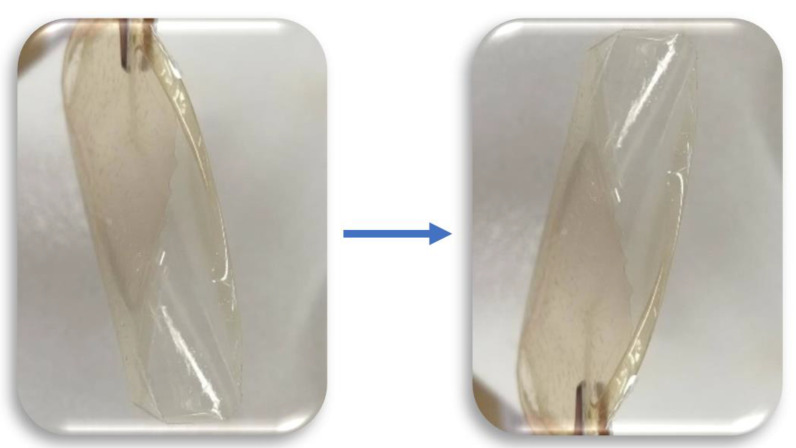
Representation of membrane bending used for determination of its malleability.

For the malleability, it was considered acceptable malleability when the membrane could bend at 360°, 4 to 9 successive times (Figure 13), without breaking and good malleability when it could bend in the same manner at least 10 times.

- Thickness

The study on the thickness of the membranes was carried out using a digital caliper, performing 8 random measurements for each membrane, with approximately 300 mg. Then the mean and standard deviation were calculated.

- Fourier Transform Infrared Spectroscopy Analysis (FTIR)

In order to verify whether the various expected components constituted the synthesized material, infrared spectra were performed using a Fourier transform attenuated total reflectance (ATR-FTIR) spectrophotometer, a Bruker FT-IR System Tensor 27 with diamond ATR crystal, in a reading range of 600 to 4000 cm^−1^. The reference spectrum (“blank”) used to perform the membrane spectra was the air itself.

- Thermogravimetric Analysis (TGA)

Each membrane was analyzed by thermogravimetry, under conditions of a ramp of 10 °C per minute, from room temperature to a temperature of 800 °C, under nitrogen flow.

It was possible to quantify the biopolymer through the following system of Equation (2):

(2) $$\{\begin{array}{c} \;weight\;lost_{polysaccharide}=x.w_{polysaccharide}+y.w_{PSil}\\ total\;weight=w_{polysaccharide}+w_{PSil} \end{array}$$

where *x* represents the biopolymer fraction and *y* the polysiloxan fraction. The total mass corresponds to the mass placed in the melting pot; the lost mass is the sum of all mass losses throughout the analysis, subtracting the initial mass loss, up to around 100 °C, which corresponds to the loss of water.

In order to obtain guidance values applicable to TGA results, the theoretical mass percentage of each component expected in the final composite was calculated. For that, Equation (3) was used:

(3) $$\%W_{A}=\frac{w_{A}}{w_{A}+w_{B}+\ldots }\times 100$$

where *W_A_* represents the mass of component A and *W_B_* the mass of component B and so on.

For this calculation, only the solutes present in the solution were taken into account, namely, the mass of polysiloxan obtained by converting the number of moles of TEOS into moles of silica and then determining the corresponding mass, the mass of chitosan and in the respective membranes, the mass of chondroitin sulfate, fucoidan or ulvan, the mass of glycerol and finally glutaraldehyde. 

#### 3.2.3. Development of Sensing Membranes for Al(III)

Obtaining a sensing membrane at an optical level involves the incorporation of a chromophore in a support material. In this article, the developed membranes acted as support material for the signaling element, the chromophore. The chromophore produces an optical signal with the color change due to the formation of a complex with Al(III).

- Analysis on the UV-Vis Spectrophotometer

**Figure 14:**
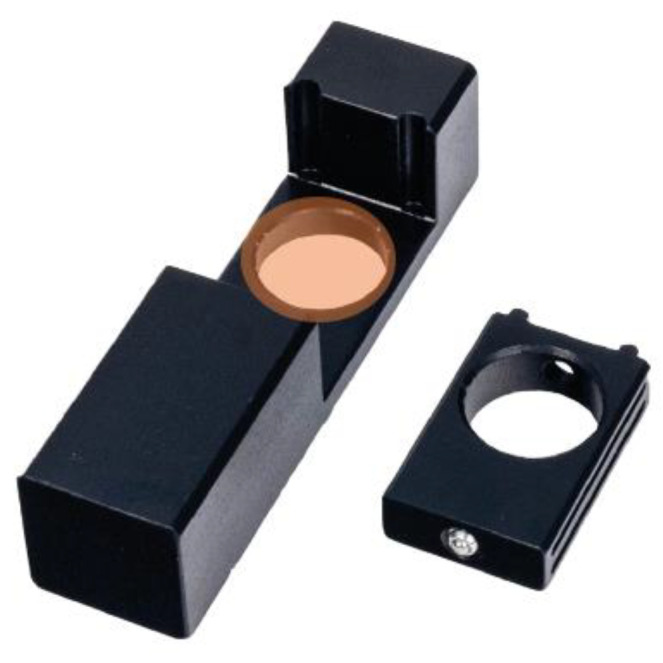
Image of the solid sample holder utilized for membrane analysis.

For the analysis of the optical changes in the membrane, UV-Vis spectrophotometry was used. To record the absorption spectra in the membranes, the analyses were carried in a PG instruments UV-Vis spectrophotometer, T60. The spectra were obtained in a range from 380 to 700 nm. In order to analyze membranes in the UV-Vis spectrophotometer a piece of the membrane was placed in a special cell (Figure 14). Then the membrane was analyzed, previous to immersion on the Al(III) solution, to register the spectrum, which was set as the reference spectrum. The membrane was then immersed in the different concentration Al(III) solutions for the time chosen and analyzed.

- Optimization of ECR Concentration

The incorporation of the chromophore was carried out by immersing 150 mg of the membrane in 10 mL of aqueous solution of ECR. In order to study the optimal concentration of ECR, different concentrations between 0.05 mmol L^−1^ and 0.5 mmol L^−1^ were tested for a period of 24 h of immersion. For this study a different piece of the same membrane was immersed on the solutions with different concentrations of ECR.

From this point on, all Al(III) solutions were prepared from the solubilization of aluminum salt in 0.002 mol L^−1^ acetate/acetic acid buffer at pH 5.5.

After ECR incorporation, the color change after a 15 min immersion period with a 10 mg L^−1^ aluminum solution was analyzed.

- Optimization of Immersion Time in Metal ion Solutions

The immersion time of ECR-incorporated membranes in metal ion solution was optimized by immersing them in a 10 mg L^−1^ solution of aluminum cation. A single piece of each tested membrane was used. The immersion time varied from 1 to 40 min. The sensing membrane was subjected to immediate analysis.

- Sensing Membrane Calibration

The ECR-incorporated membrane before immersion in the metal ion solution was used as reference spectrum. Subsequently, the same membrane was subjected to immersion in different concentrations of metal ion solution (from 0.1 to 5 mg L^−1^) for regular intervals of 30 min and the spectrum was obtained for each case. The UV-Vis spectra obtained were analyzed using the OriginPro9 program [26]. Peaks and baseline analysis were carried out (as represented in Figure S7) to find the absorbance maxima and corresponding wavelengths.

- Study of the Effect of Surfactants

The incorporation of surfactants into the membrane was expected to enhance the optical performance of the sensing membranes. Thus, the incorporation of the cationic surfactant cetyltrimethylammonium bromide, CTAB, was carried out and analyzed in this study. 

An approach where the surfactant was incorporated into the test solution was chosen. The surfactant was added to the analyte solution (2 mL of 1 mmol L^−1^ surfactant solution to 10 mL of the metal cation solution in acetate buffer of pH 5).

- Detection Limit Determination

The detection limit was determined using Equation (4):

(4) $$L.D.=\frac{s\times 3.3}{m}$$

where *s* represents the standard deviation determined from the analysis of several “blanks” and *m* the slope of the respective calibration curves.

The “blank” standard deviation was achieved by performing the baseline in a portion of the membrane and subsequent analysis of different portions of the membrane.

- Study on the Effect of Interfering Species

The potential ions interfering with Al(III) such as Cu(II), Fe(II), Fe(III), Cd(II), Pb(II), Cr(VI) and F^−^ were selected. Their interfering effect was studied by immersing the membrane for 30 min in a solution containing fixed concentration of Al(III) ions along with varying concentrations of interfering ions. The study was conducted using the same piece of the membrane when changing the concentration, going from the lowest to the highest concentration of the same interfering species. Different pieces of the membrane were used for the different interfering species. From the data obtained, the selectivity coefficient was calculated using Equation (5).

(5) $$K_{Al,\;Int}=\left(A_{Int}/C_{Int}\right)/\left(A_{Al}/C_{Al}\right)$$

where *A_Int_* and *A_Al_* stand for absorbance of interferent and Al(III) at corresponding concentrations *C_Int_* and *C_Al_*.

## 4. Conclusions

The membranes were synthetized by using biopolymers extracted from by-products and wastes of marine-related activities, as major components. These materials are biocompatible and biodegradable, which is important to lead to a low ecologic impact.

The use of sulphated polysaccharides proved to be essential to improve the optosening membranes. Due to its stronger interaction with the metal cations, more of these cations permeate the membrane, allowing it to form more complexes of chromophore—metal cation—which leads to an increase in the intensity signal given by the complex formation. The presence of the sulphated polysaccharides in the membranes gave substantially lower detection limits compared to their absence. From all the sulphated polysaccharides, ulvan was the one that outperformed all others, giving the best results.

In the case of selectivity, the use of the surfactant CTAB was shown to be essential to detect aluminum in the presence of other interfering species, specially Cu(II), which seemed to be the most competitive among many other cations. The formation of ternary complexes between the surfactant monomers, chromophore and aluminum cation led to an improvement in sensitivity and red shift, which contributed to a better detection of this cation when in solution with the presence of other competitive cations, such as Cu(II).

These membranes present a significant potential for improvement in the performance for the analysis of Al(III) and probably other metal cations. Natural waters and other simple uncolored matrices are among the types of samples amenable to the application of these membranes into optical probes for in situ or lab analysis of toxic heavy metals, in a cheap and quick way. Although the sulphated polysaccharides can be marketed at much higher prices compared to chitosan, the small contents required in the formulations should not inhibit an eventual commercialization, especially if the expected performance gains are accounted for.

## 5. Patents

Pending Patent #116976 (Portugal) to Universidade do Porto|SARSPEC, LDA.

## Data Availability

Not applicable.

## Supplementary Materials

The following supporting information can be downloaded at: https://www.mdpi.com/article/10.3390/molecules27155026/s1, Figure S1: Graphical representation of DTG for CH-PSil membranes and individual polysaccharides; Figure S2: Graphical representation of DTG for CH-CS membranes and individual polysaccharides; Figure S3: Graphical representation of DTG for CH-Fu membranes and individual polysaccharides; Figure S4: Absorbance spectra of different membranes with different immersion times in 10 mg L^−1^ Al(III) solution for kinetic studies; Figure S5: Absorption spectra of the different types of sensing membranes in solutions with different Al(III) concentrations with CTAB; Figure S6: Graphical representation of the UV-Vis spectra of the membrane CH-CS after immersion in different concentrations of: (a) Fe(II); (b) Fe(III); (c) Cd(II); (d) Pb(II); (e) Cr(VI) e (f) F^−^; Figure S7: Representation of baseline selected on the OriginPro 9 and the peak selection.
