# Supplementary material for: Improved Metal Cation Optosensing Membranes through the Incorporation of Sulphated Polysaccharides
Source: Molecules. 2022 Aug 7;27(15):5026. doi: 10.3390/molecules27155026 (PMC9370371; doi:10.3390/molecules27155026)
Supplement: Supplementary file 1 [file molecules-27-05026-s001.zip › molecules-1829317-supplementary.pdf]

Improved metal cation optsosensoring membranes through the incorporation of sulphated polysaccharides

P. R. M. Santos[a], A. Johny[a], C. Q. Silva[a,b], M. A. Azenha[a,*], J. A. Vázquez[c], J. Valcarcel[c], C. M. Pereira[a] and A. F. Silva[a]

*Corresponding author: Tel.: +351 220402628; Fax.: +351 220402659. Email: mazenha@fc.up.pt

Supplementary Information

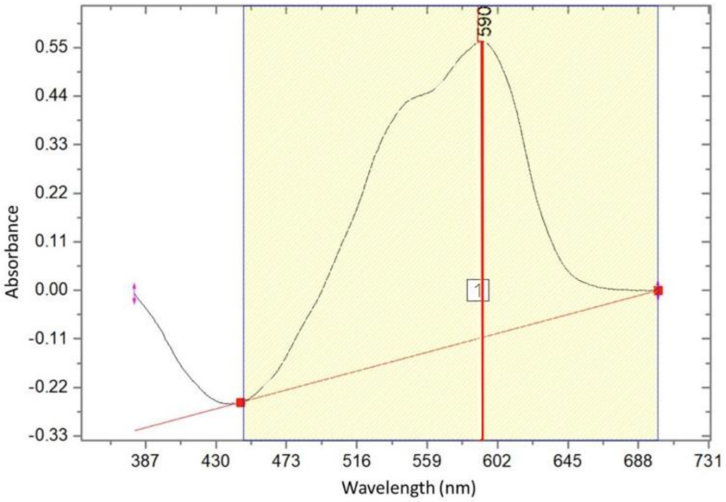

Figure S1: Representation of baseline selected on the OriginPro 9 and the peak selection. The represented spectrum was obtained by using the ECR-loaded membrane as reference. The increase of Al(III) concentration implicates the decrease of free ECR, what explains the “negative band” observed at the ECR band region. Comparing with other possible spectra processing methods, this provided the best calibration curves (higher signals and lower concentration readability).

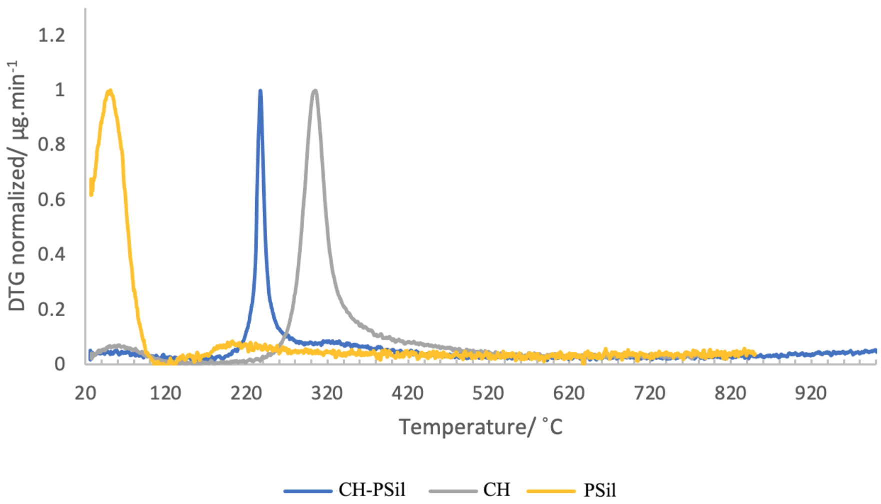

Figure S2: Graphical representation of DTG for CH-PSil membranes and individual polysaccharides

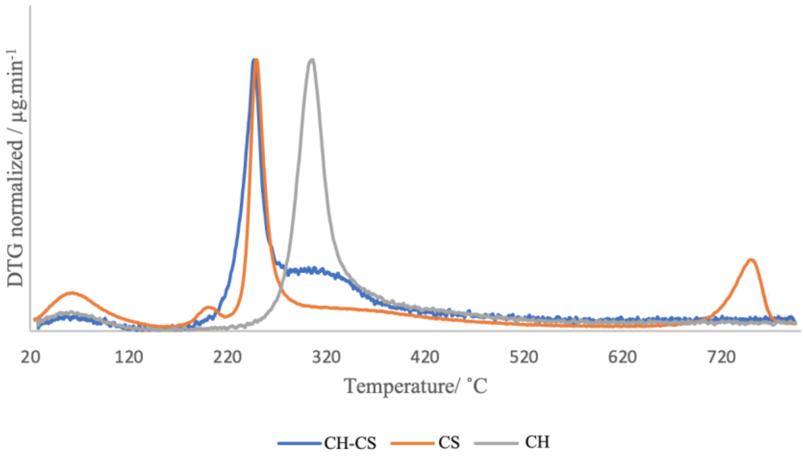

Figure S3: Graphical representation of DTG for CH-CS membranes and individual polysaccharides

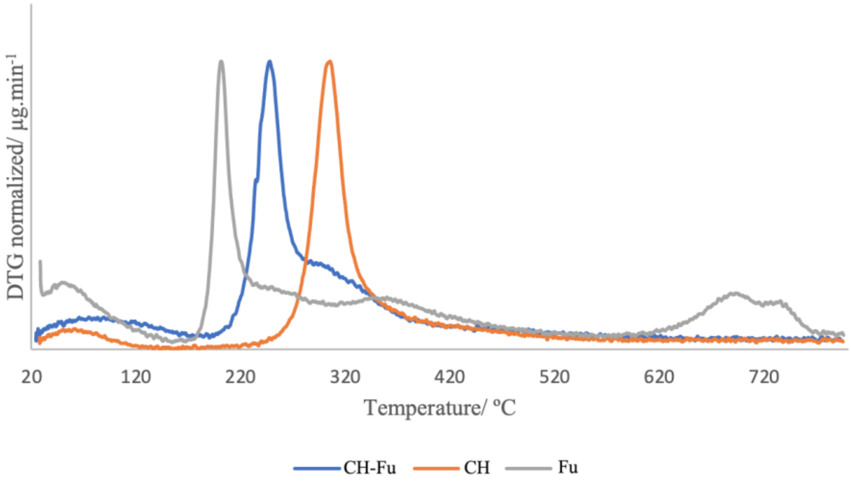

Figure S4: Graphical representation of DTG for CH-Fu membranes and individual polysaccharides

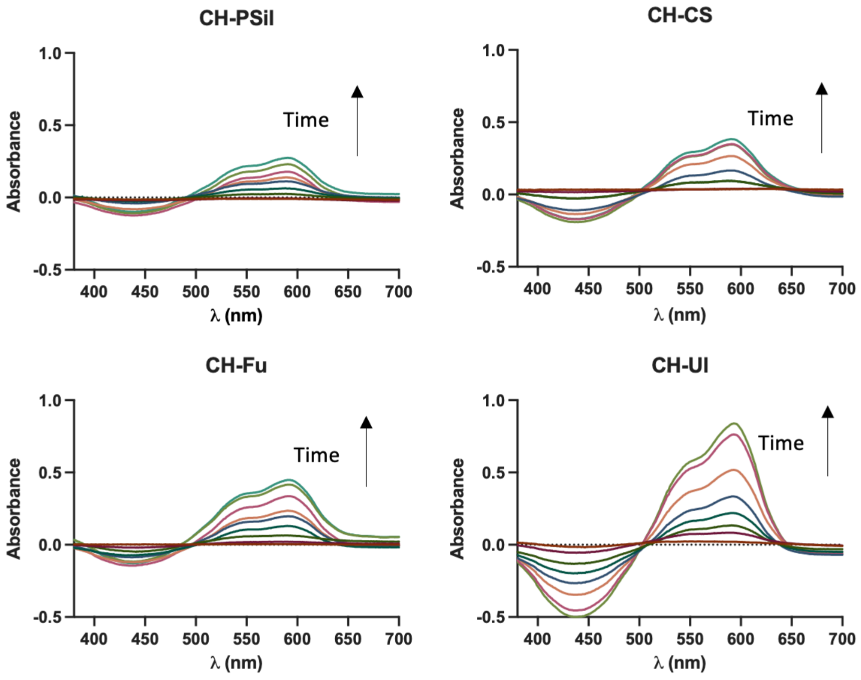

Figure S5: Absorbance spectra of different membranes with different immersion times in 10 mg·L^{-1} Al(III) solution for kinetic studies

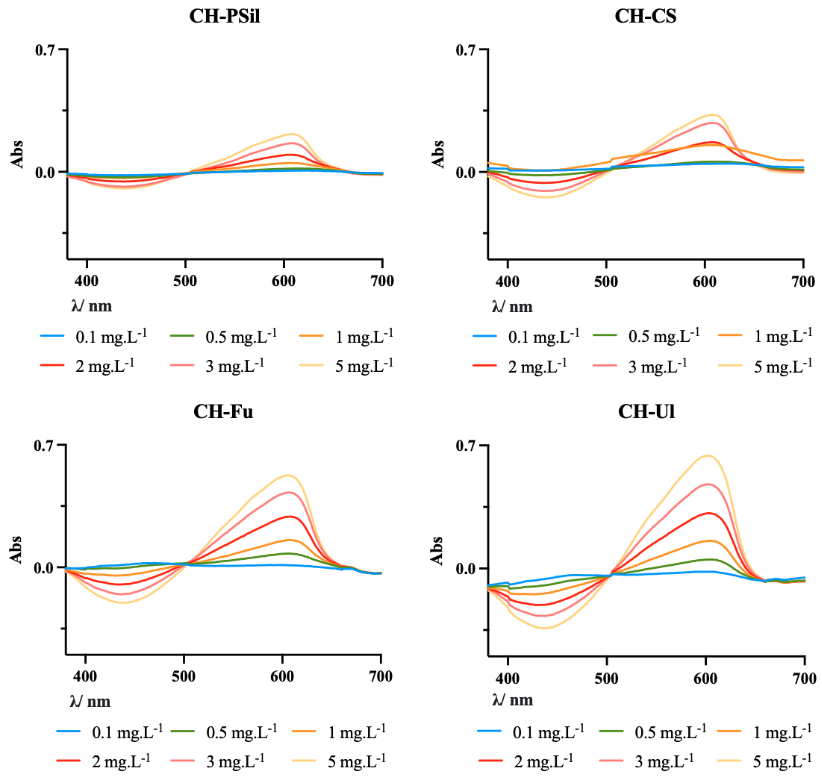

Figure S6: Absorption spectra of the different types of sensing membranes in solutions with different Al(III) concentrations with CTAB

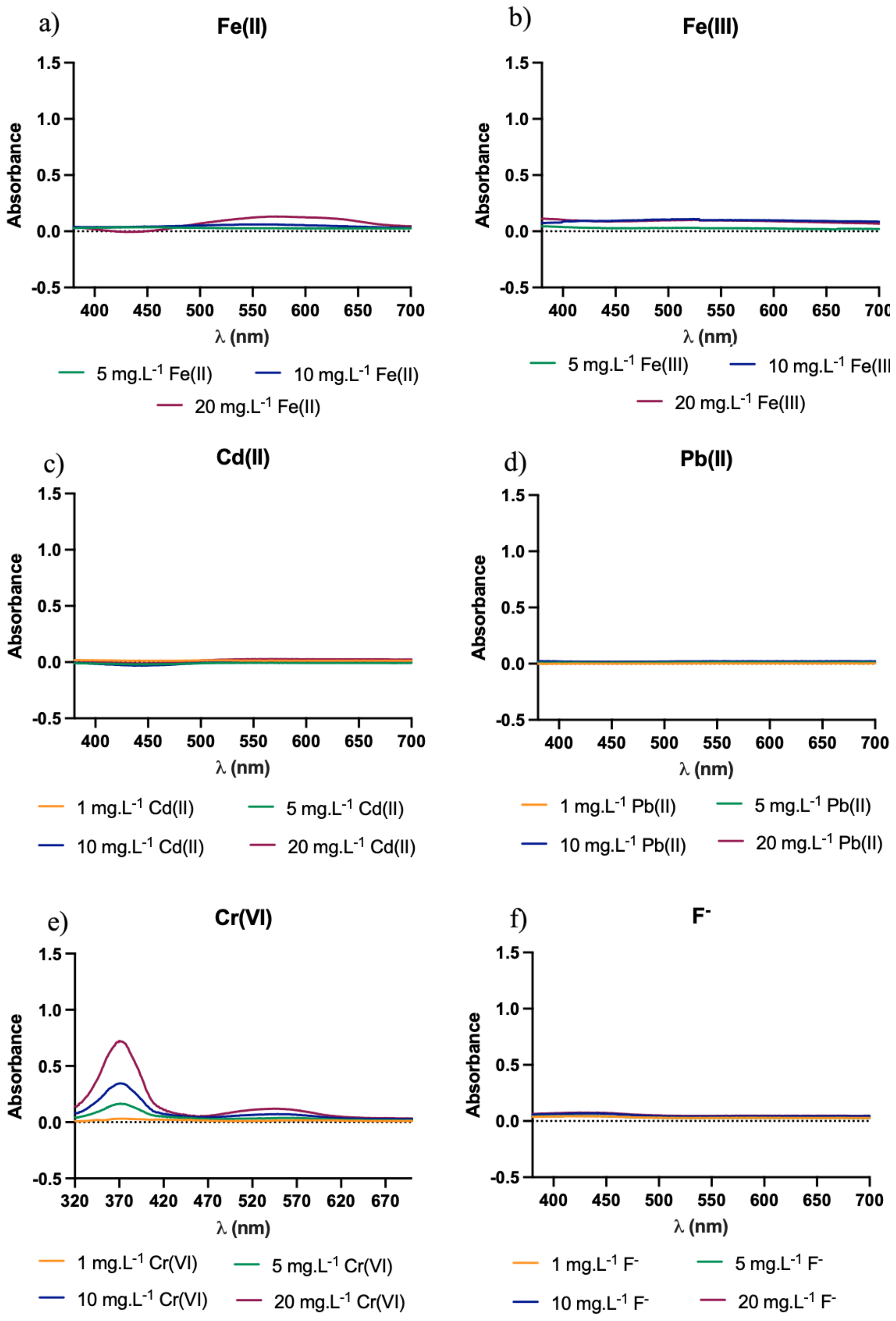

Figure S7: Graphical representation of the UV-Vis spectra of the membrane CH-CS after immersion in different concentrations of: a) Fe(II); b) Fe(III); c) Cd(II); d) Pb(II); e) Cr(VI) e f) F^-.
